# Optimizing stent retrievers for mechanical enhancement and in vitro testing in acute ischemic stroke models

**DOI:** 10.1186/s12938-024-01312-1

**Published:** 2024-11-27

**Authors:** Jae-Won Lee, Han-Ki Kim, JinWoo Kim, Hyuk Choi

**Affiliations:** 1 Department of Convergence Medicine, College of Medicine, Korea University, Seoul, South Korea; 2 Department of Bionanosystem Engineering, Graduate School, Jeonbuk National University, Jeonju, South Korea

**Keywords:** Stent retriever, Acute ischemic stroke, Stent design, Finite-element analysis, Performance evaluation

## Abstract

**Background:**

Acute ischemic stroke (AIS) remains a major cause of morbidity and mortality worldwide. Mechanical thrombectomy, especially with stent retrievers, offers a promising treatment, particularly for patients ineligible for intravenous tissue plasminogen activator (IV tPA) therapy. This study aimed to develop and evaluate novel stent retriever designs to enhance mechanical properties and vessel compatibility.

**Results:**

We evaluated four stent designs using finite-element analysis (FEA) to assess their mechanical properties. Based on these evaluations, Stent D emerged as the optimal design due to its superior elasticity and adaptability. Comparative testing of Stent D against commercial stents, Solitaire FR and Trevo XP ProVue, revealed the following metrics: radial forces of 3.77 ± 0.01 N for Solitaire FR, 3.92 ± 0.08 N for Trevo XP ProVue, and 4.10 ± 0.07 N for Stent D; flexibility measurements of 0.38 ± 0.11 N for Solitaire FR, 0.91 ± 0.11 N for Trevo XP ProVue, and 0.59 ± 0.05 N for Stent D; deployment forces of 0.37 ± 0.02 N for Solitaire FR, 0.42 ± 0.04 N for Trevo XP ProVue, and 0.32 ± 0.02 N for Stent D; and recapture forces of 0.38 ± 0.01 N for Solitaire FR, 0.45 ± 0.02 N for Trevo XP ProVue, and 0.35 ± 0.01 N for Stent D. Thrombus retrieval rates were 96.16% for Solitaire FR and 95.51% for Stent D.

**Conclusions:**

These findings demonstrate that Stent D performs comparably to commercial stents, highlighting its effective performance in AIS treatment. Stent D shows promise as a candidate for further clinical evaluation due to its superior mechanical properties and effective thrombus retrieval capabilities.

## Background

**Fig. 1:**
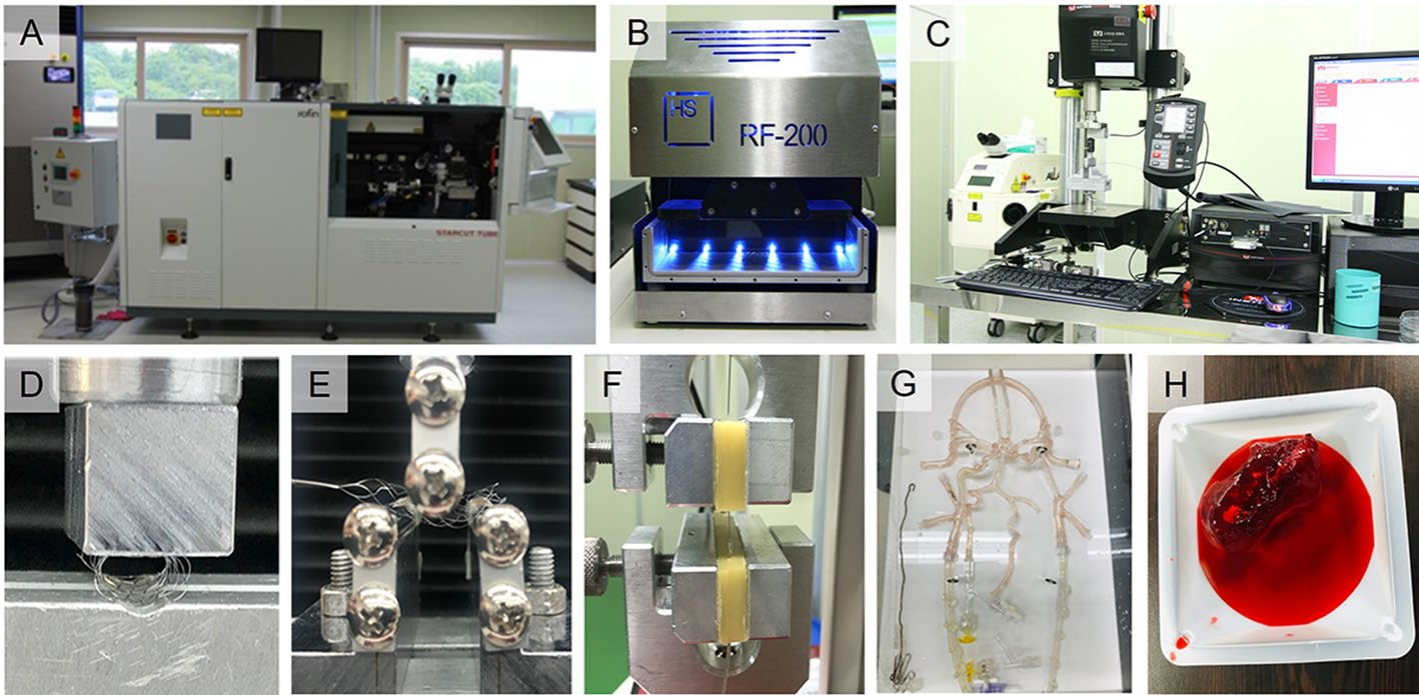
**Experimental apparatus**. **A** Laser tube cutting machine, Starcut tube fiber (Rofin-Baasel Lasertech). **B** Radial force test device, RF-200 (Heinz Schade GmbH). **C** Micro-universal testing machine, T0-100S (Test One). **D** Radial force test jig. **E** Flexibility test jig. **F** Deployment and recapture test jig. **G** Cerebrovascular phantom. **H** Artificial thrombus

Acute ischemic stroke (AIS) remains a major cause of morbidity and mortality worldwide, prompting continuous efforts to develop effective treatment strategies [1, 2]. While intravenous tissue plasminogen activator (IV tPA) therapy has demonstrated efficacy in thrombolysis within a limited time window, its application is constrained by factors such as treatment eligibility criteria and the risk of hemorrhagic complications [3, 4]. In recent years, mechanical thrombectomy has emerged as a promising alternative or adjunctive therapy for AIS, particularly for patients with large vessel occlusions or those ineligible for IV tPA [5, 6]. Among the various mechanical thrombectomy techniques, stent retriever deployment has garnered significant attention due to its ability to achieve rapid and complete recanalization of occluded vessels [7, 8]. However, current stent retriever designs, exemplified by the Solitaire FR (Medtronic, USA) and Trevo XP ProVue (Stryker, USA) devices, possess inherent limitations. These devices, composed of nitinol alloy tubes and fabricated using laser-cutting techniques, typically feature a closed-cell design that may compromise flexibility and conformability within the vasculature compared to open-cell designs [9, 10]. Given these challenges, there is a growing interest in developing novel stent retriever designs that optimize mechanical properties while enhancing vessel compatibility. This study aims to address this need by introducing four innovative stent retriever prototypes. Through comprehensive evaluation utilizing finite-element analysis (FEA) and bench-top testing (Fig. 1), we seek to identify the most promising design candidate that not only improves recanalization rates but also minimizes the risk of vascular injury and procedural complications. Following optimization, the performance of the stent was compared with that of the two products.

## Results and discussion

### Stent elasticity and adaptability using the computational solid mechanics

**Fig. 2:**
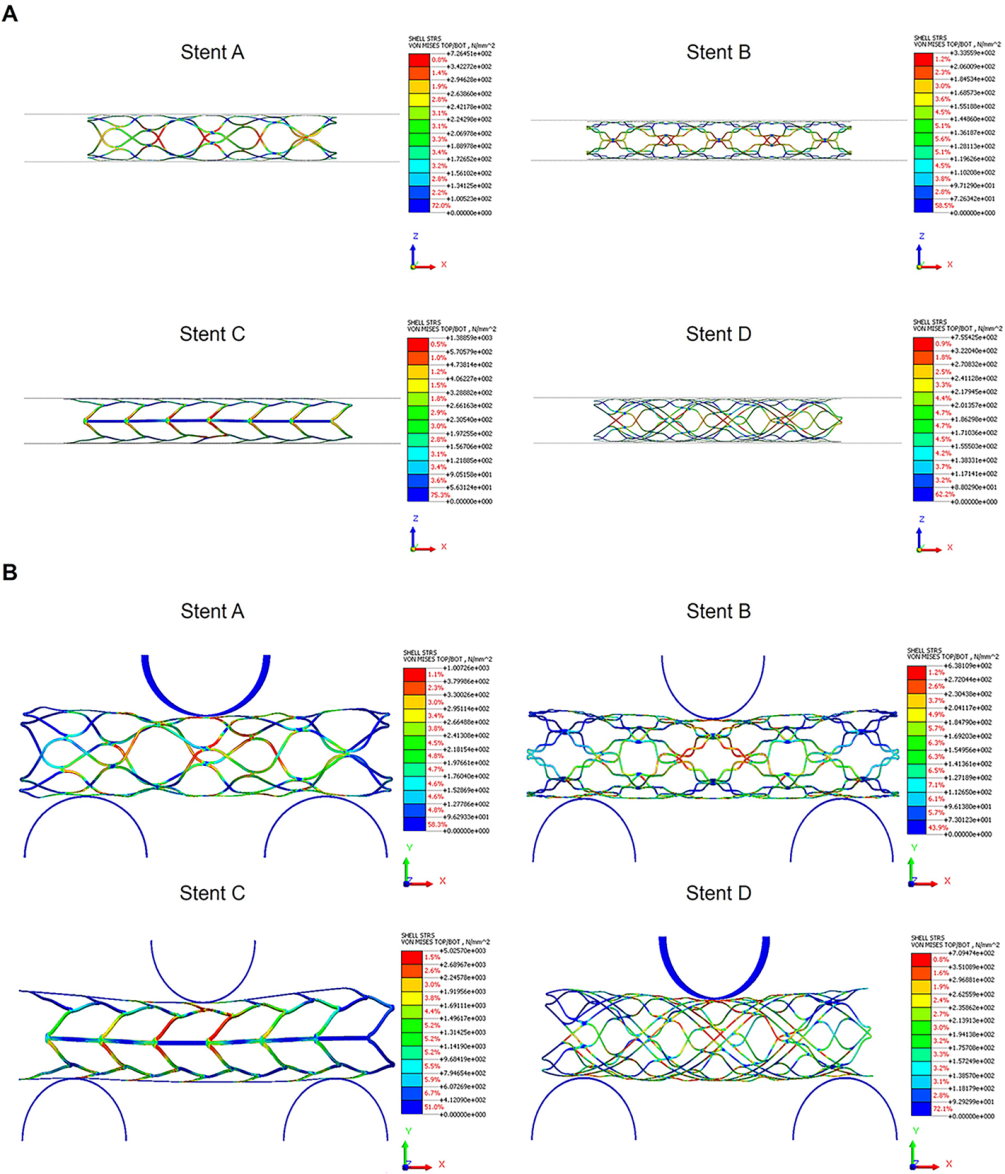
**FEA Results of stent elasticity and adaptability**. **A** Flat type Radial force test of stents: Stent was placed in between the top and bottom plate and the top plate was compressed to give a load up to 50% (2.2 mm) of the radius of stent. The radial force of stent A, stent B, stent C, and stent D were 0.360, 0.184, 1.106, and 0.330 N, respectively. **B** 3-point bending test of stents: The result of 3-point bending test when giving 6.6-mm forced displacement to each stent models was 0.286, 0.268, 0.356, 0.098 N, respectively

Stent elasticity and adaptability were assessed using Midas NFX software, employing computational solid mechanics. To validate the stent test results, the FEA model's elasticity and adaptability were evaluated through flat-type radial force tests and three-point bending tests, designed to simulate real-world conditions [11, 12]. Figure 2 presents the evaluation of the radial force following the application of the flat-type radial force test. Compression was performed by positioning the stent between upper and lower plates and applying a load equivalent to 50% (2.5 mm) of the stent's radius. Radial forces for stent A, stent B, stent C, and stent D were 0.360, 0.184, 1.106, and 0.330 N, respectively (Fig. 2a). Stent C showed the highest expansion force; However, such excessively high radial force stents can lead to severe adverse events more frequently, including vessel damage, restenosis, and even hemorrhages [13, 14]. the reaction force values measured following the application of an external load using a three-point bending test. A displacement of 3.2 mm was applied to the top load applicator, and the reaction force of each stent model was measured. Reaction forces for stent A, stent B, stent C, and stent D were 0.242, 0.166, 0.296 N, and 0.226N, respectively (Fig. 2b). Likewise, Stent C showed the highest reaction force, and stent B, the lowest. Although Stent C showed higher reaction forces, indicating greater rigidity, this could potentially translate to lower flexibility and adaptability in tortuous vessel anatomies. Conversely, Stent B's lower reaction force in the bending test suggests it is more flexible, which is beneficial for navigating complex vascular pathways without causing additional trauma or vessel injury. A balance between radial force and flexibility is necessary to minimize these risks while ensuring the stent can adequately support the vessel walls [15, 16]. Stent D demonstrated a balanced combination of adequate elasticity and adaptability, which enhances safety and therapeutic outcomes in cerebrovascular applications.

### Structural performance comparative evaluation

**Fig. 3:**
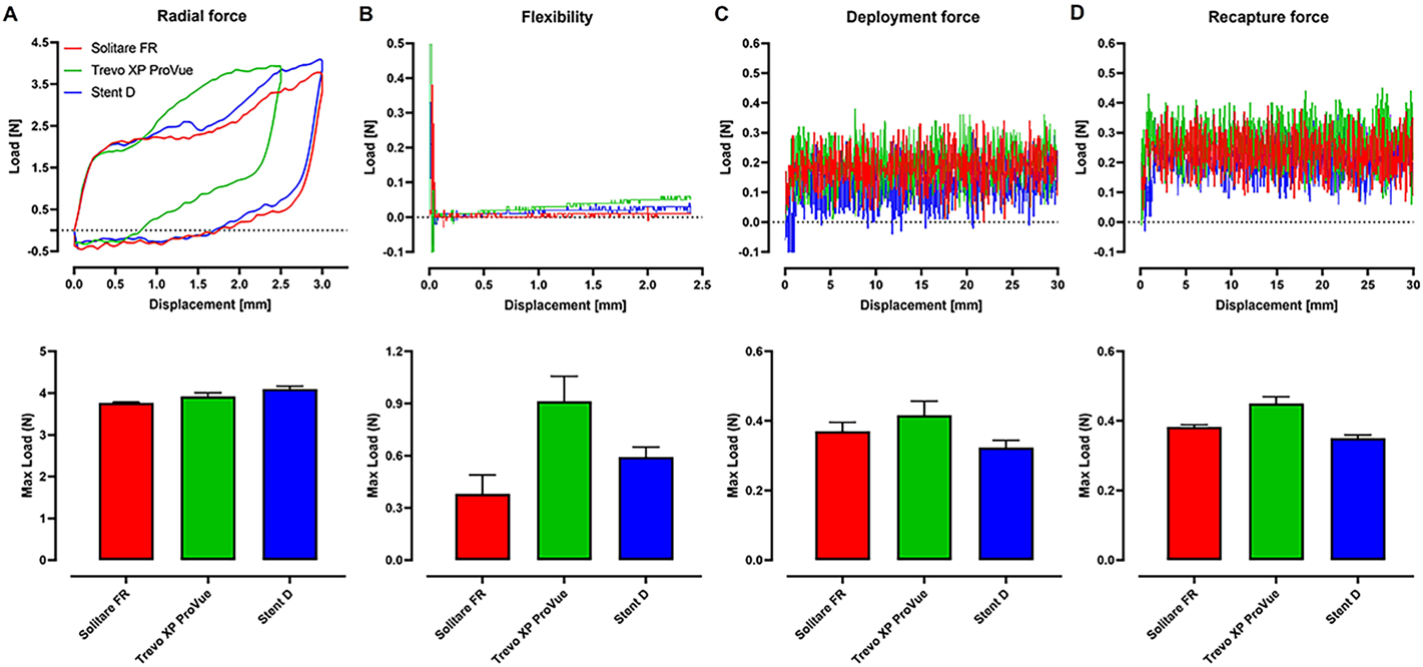
**Comparative performance evaluation of structural features**. **A** Radial force, **B** flexibility, **C** deployment force, and **D** recapture force. All tests were conducted with n = 3 replicates

In Fig. 3 and Table 1, to compare the structural performance of stents, we evaluated the most critical factors of radial force, flexibility, deployment force, and recapture force against commercially available products in Korea, including Solitaire FR and Trevo XP ProVue.

**Table 1:** Actual measurement values of comparative evaluation

	Solitare FR	Trevo XP ProVue	Stent D
Radial force (N)	3.77 ± 0.01	3.92 ± 0.08	4.10 ± 0.07
Flexibility (N)	0.38 ± 0.11	0.91 ± 0.11	0.59 ± 0.05
Deployment force (N)	0.37 ± 0.02	0.42 ± 0.04	0.32 ± 0.02
Recapture force (N)	0.38 ± 0.01	0.45 ± 0.02	0.035 ± 0.01

Radial force is crucial, because insufficient radial force can lead to deformation of the stent, preventing it from expanding the vessel properly and fully removing the thrombus, which can result in secondary occlusion [14, 17]. Radial force measurements were conducted using Heinz's RF-200 (Heinz Schade GmbH, Mittelstadt, Germany) equipment to assess flat radial force. The stents were compressed by applying force from the top plate via a load applicator until they reached 50% of their diameter, and the reaction force was measured after achieving stent displacement. The results of the radial force examination indicated that Stent D exhibited the highest values: 3.77 ± 0.01, 3.92 ± 0.08, and 4.10 ± 0.07 N for Solitaire FR, Trevo XP ProVue, and Stent D, respectively, as depicted in Fig. 3a and Table 1. The high radial force of Stent D indicates its ability to maintain shape within the vessel, ensuring effective expansion and better apposition to the vessel wall. This is critical for preventing thrombus migration and securely capturing and removing the thrombus.

Flexibility is critical for the stent's ability to navigate through tortuous and curved vascular pathways. If the stent lacks flexibility and exerts excessive force to revert to its original straight shape, it can cause vessel injury and subsequent inflammatory responses, leading to restenosis [17]. Flexibility tests were conducted using the three-point bending test according to ASTM F2606-08 standards, and force measurements corresponding to the bending displacement of the thrombectomy device were obtained using a Micro-Universal Testing Machine (UTM) system (T0-100S, Test One, Republic of Korea). The results in Fig. 3b and Table 1 showed respective values of 0.38 ± 0.11 N for Solitare FR, 0.91 ± 0.11 N for Trevo XP ProVue, and 0.59 ± 0.05 N for Stent D. Since lower values indicate higher flexibility, this suggests better flexibility in the order of Solitaire FR, Stent D, and Trevo XP ProVue. It was observed that during the initial phase of the three-point bending test, there was a sharp increase in load, followed by a significant drop. This initial peak in force can be attributed to the contact between the stent and the testing apparatus, where the initial resistance to deformation is encountered. However, as the bending continued, the force required decreased substantially, likely due to the material properties of the nitinol stents, which exhibit superelasticity and nonlinear deformation characteristics [18–20]. Accurately capturing these nuanced mechanical behaviors requires high-resolution load cells, capable of detecting subtle variations in force throughout the deformation process. Unfortunately, most UTM systems may not possess the necessary sensitivity to fully capture the low-force, high-deformation characteristics of nitinol stents, leading to potential underestimation of their performance in a clinical setting. This limitation should be considered when interpreting the results, as it may affect the precision of the measurements and the overall assessment of stent flexibility and resilience. Closed-cell stents, characterized by their interconnected struts, offer greater radial support but are inherently less flexible compared to open-cell stents. Open-cell stents, with their segmented structures, provide enhanced axial flexibility, allowing them to better conform to the intricate and curved pathways of blood vessels [21]. Stent D features a hybrid structure that combines both open and closed cells. This hybrid design effectively balances axial flexibility with radial stiffness, ensuring that the stent can navigate through complex vessel anatomies without causing damage, thereby reducing the risk of post-procedural complications such as restenosis.

Deployment force is the force required to advance the stent to the target site. A low deployment force is crucial, because it helps the device penetrate the clot effectively and quickly upon deployment, improving the chances of successful clot removal with fewer attempts while protecting the blood vessel walls from damage [22]. The evaluation method for deployment force involved securing the catheter to the UTM and utilizing a compression module to push the thrombectomy device out of the catheter, measuring the force generated in this process. In Fig. 3c and Table 1, the respective values were 0.37 ± 0.02 N for Solitaire FR, 0.42 ± 0.04 N for Trevo XP ProVue, and 0.32 ± 0.02 N for Stent D. Unsuccessful stent delivery to the target lesion has been associated with both micro and macroscopic damage to the stent structure. Stent D exhibited a moderate deployment force, ensuring ease of use during the procedure without compromising the integrity of the catheter or the device, thus facilitating smoother and safer stent delivery.

Recapture force is the force required to retract the thrombectomy device back into the catheter after it has been deployed, which is critical for repositioning or removing the device. High recapture force can hinder procedural efficiency and pose risks of the stent detaching from the catheter. For recapture force assessment, the catheter with the thrombectomy device deployed to approximately 50% was secured to the UTM, and a tension module was used to pull the thrombectomy device back into the catheter, measuring the force generated during this process. The results in Fig. 3d and Table 1 showed respective values of 0.38 ± 0.01, 0.45 ± 0.02, and 0.035 ± 0.01 N. Stent D's intermediate recapture force ensures that the stent can be easily repositioned or retrieved without considerable difficulty, thereby improving overall procedural safety and flexibility.

This study comparatively analyzed the performance of three stents using universally recognized performance testing methods. The performance of Stent D observed in the tests is crucial for achieving successful clot removal with minimal attempts, demonstrating the potential to reduce procedure time and minimize risks associated with vascular manipulation.

### Thrombus retrieval rate evaluation

**Fig. 4:**
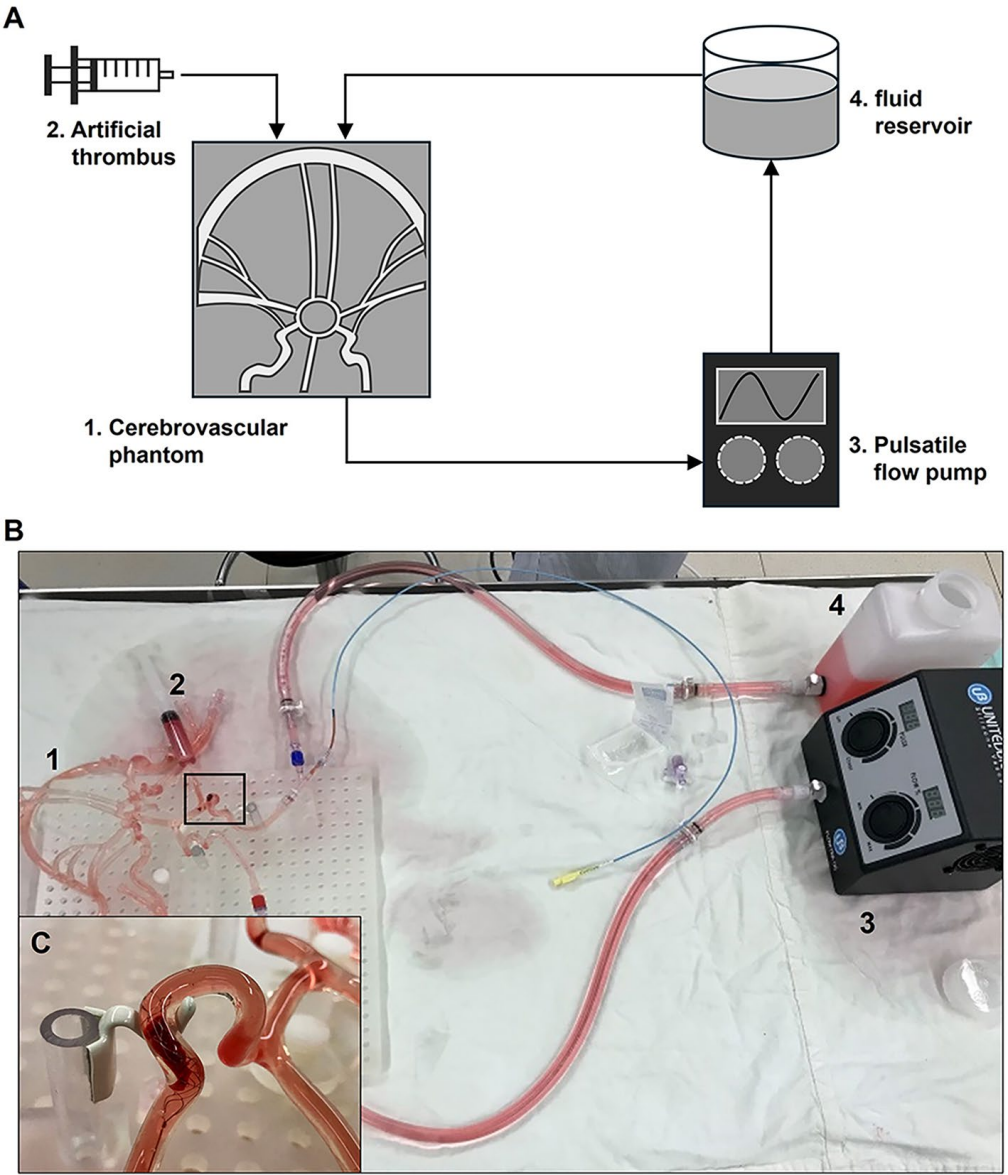
**Schematic and experimental setup for thrombus retrieval rate evaluation using a cerebrovascular phantom model**. Schematic representation (**B**) and detailed visualization (**A**) of the experimental setup: (1) cerebrovascular phantom capable of thrombus loading (**C**). (2) Artificial thrombus composed of 88.9% PAAM and 93% solvent (DI water, 100 wt%). (3) Pulsatile flow pump, FlowTek125 (United Biologics, Inc.), and (4) fluid reservoir

Following mechanical thrombectomy, patients with AIS may experience secondary occlusion of the cerebral artery due to incomplete thrombus removal, which can subsequently lead to occlusion of smaller vessels [23]. To evaluate the effectiveness of the stents in thrombus retrieval, we used AIS model with cerebrovascluar phantom and artificial clots made from polymer hydrogel. The experimental setup, shown in Fig. 4, includes a cerebrovascular phantom capable of simulating the vascular conditions, an artificial thrombus composed of 88.9% PAAM and 93% solvent (100 wt% DI water), a pulsatile flow pump (FlowTek125, United Biologics, Inc.), and a fluid reservoir. The pulsatile flow pump replicates physiological blood flow, allowing us to assess the stent’s performance under conditions that mimic real blood flow dynamics. This setup enabled us to conduct three mock procedures to determine the efficiency of the stents in retrieving thrombi [24, 25]. The thrombus retrieval rate was calculated as the percentage reduction in thrombus weight before and after the intervention using the following equation:

$$Recovery\; rate\, (\%)= \frac{Pre-Intervention\; Clot\; weight\, (g)}{Post-Intervention\; Clot\; weight\, (g)} \times 100$$

The results for In vitro blood clot removal rate measurement are presented in Table 2. The comparison of blood clot removal rates between Solitare FR and Stent D revealed a recovery rate of 96.16% for Solitare FR and 95.51% for Stent D. Both stents achieved thrombus retrieval rates of over 90% when inserted into the vascular phantom model. These findings suggest that there is no significant difference in thrombus removal performance between the two stents, indicating comparable results.

**Table 2:** In vitro blood clot removal rate measurement results

	Clot weight (g)		Recovery rate (%)
	Pre-intervention	Post-intervention	
Solitare FR	22.14	21.74	98.19
	25.92	25.64	98.92
	19.87	18.55	93.36
			96.16
Stent D	15.37	14.93	97.13
	16.99	15.54	91.47
	25.68	25.15	97.94
			95.51

## Conclusion

In this study, we proposed various shapes and designs of stent retriever for application in AIS patient models and determined the optimal stent design through FEA. After thorough analysis, Stent D emerged as the optimized design choice. This newly developed stent exhibits promising mechanical properties and structural performance, comparable to, and in some aspects superior to, existing commercially available products such as Solitaire FR and Trevo XP ProVue. Its well-balanced combination of mechanical attributes, including adequate radial force, optimal flexibility, moderate deployment force, and robust recapture force, positions it as a suitable candidate for further clinical evaluation. Moreover, the thrombus retrieval rate evaluation conducted using the vascular phantom model yielded promising results, indicating that Stent D effectively retrieves thrombi in AIS patient models. This underscores the comprehensive functionality of Stent D, not only in terms of mechanical performance but also in its ability to address the primary objective of thrombus removal.

However, despite the positive outcomes from this study, it has limitations. Since stents are implanted within the human body, mechanical properties alone are not sufficient considerations. A limitation of this study is the absence of biocompatibility evaluation. Therefore, in future studies, we plan to conduct biocompatibility tests in accordance with ISO 10993 standards to ensure the cytocompatibility of our stent design. Specifically, we will investigate methods such as electropolishing and surface coatings with materials like oxide films and hyaluronic acid to improve the biocompatibility of the nitinol stents. These future investigations will provide a comprehensive understanding of Stent D's safety and effectiveness in vivo, paving the way for larger-scale clinical trials and extended follow-up studies that are crucial to fully establish its clinical efficacy and safety in thrombectomy procedures.

## Methods

### Model geometry

**Fig. 5:**
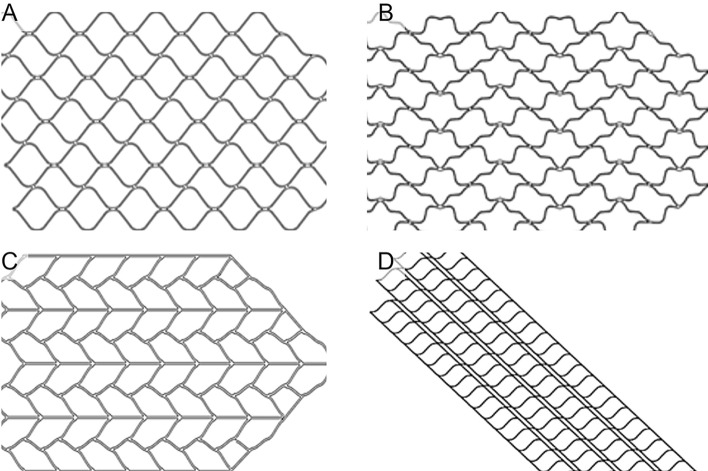
**Model geometry of stent:** **A** stent A, **B** stent B, **C** stent C, and **D** stent D. Strut was manufactured with thickness of 0.08 mm, width of 0.075 mm, length of 0.15 mm and 8 bands

Three-dimensional (3D) computational models were designed using CATIA P3 V5R18 (Dassault Systems, Vélizy-Villacoublay, France) software resulting in four types of stents, namely stent A, stent B, stent C and stent D. Nitinol tubes for stent manufacturing was purchased from Vascotube GmbH (Germany) with 5.0 mm of outer diameter and 0.08 mm of wall thickness. Stents were designed to prevent intraluminal stent protrusion, and the strut was manufactured with a thickness, width, length of 0.08, 0.075, and 0.15 mm, respectively, containing eight bands. Our stent design was based on the well-known clinically effective Enterprise stent. Figure 5 shows the four stent models designed. Stent A was designed with alternating square and rectangular arrangements, utilizing S-shaped linkers (Fig. 5a). Stent B modified the typical diamond strut structure into an S-shape (Fig. 5b). Stent C arranged leaf-like shapes in a zig-zag pattern, connected by straight peak-to-valley linkers (Fig. 5c). Stent D featured a hybrid cell structure by adding open cells to the design of Stent A (Fig. 5d).

### Computational solid mechanics

The elasticity and adaptability of the stents were assessed using Midas NFX software (Midas IT, London, UK), leveraging computational solid mechanics. The nonlinear static mode was chosen for the simulation to accommodate the large deformations and superelasticity exhibited by the nitinol stent during flat-type and three-point bending tests. Material properties for the nitinol stents were defined with an elastic modulus of 28 GPa, a Poisson’s ratio of 0.3, a material density of 6.45 g/cc, and a tensile strength of 100 MPa, while the jigs were modeled as rigid bodies. Stents and jigs were both meshed with hexahedral solid finite elements, with element sizes set to 0.04 mm for the stents and 0.1 mm for the jigs. To validate these assessments, flat-type radial and three-point bending tests were performed on the FEA model to replicate real-world conditions. Boundary conditions were applied by fixing the bottom plate, while the top plate was constrained in all directions except for displacement along the *y* axis, where displacement loading was allowed. During the high elasticity evaluation, stents were compressed up to 50% of their diameter by enforcing displacement through a load applicator on the top plate, with the reaction force measured after achieving a 2.5 mm displacement. Adaptability was evaluated by measuring the reaction force during the three-point bending test, where enforced displacement was applied to replicate the experimental setup. The reaction forces were recorded as the upper load applicator induced deflection in the stent.

### Manufacturing of stents

For the manufacturing of NiTi alloy stents, the standard fabrication process included laser machining, chemical treatment, and heat treatment. The NiTi alloy tubes (Johnson Matthey Inc. in San Jose, CA, USA) was machined using a photolithography-based laser processing method (Starcut tube fiber, Rofin Bassel Lasertech, Germany) with a power output of 55 W, a laser wavelength of 16.5 µm (pulse), and an oxygen cooling system. Following laser cutting, chemical etching was performed, which involved two steps: cleaning and etching. The primary goal of chemical polishing was to eliminate impurities, defects, residual stress, and oxide films from earlier operations. The cleaning stage, lasting 30 min at room temperature, focused on removing oxides and organic materials. In addition, a deoxidizing process lasting 30 s at room temperature was conducted to remove oxide layers. Organic residues on the nitinol stent surfaces were removed using commercial solutions, MicroClean Ti solution™ (RDZ-1849; RD Chemical, Mountain View CA, USA), followed by MicroClean MV™ (RDZ-1995; RD Chemical, Mountain View CA, USA), applied at room temperature for 30 s to eliminate the oxide film. Heat treatment was performed using a high-vacuum annealing furnace (HT-1000, Heinz Schade, Germany) to produce a stable oxide layer with the desired mechanical properties for the nitinol stents. The heat treatment parameters were selected within the range of 450–600 °C and 10–30 min [26, 27]. In this study, we chose a heat treatment condition of 510 °C for 10 min, as it resulted in the most stable oxide layers and suitable mechanical properties for the nitinol stents.

### Cerebrovascular thrombus phantom model

Computed tomography data from patients with cerebrovascular diseases were collected to fabricate a silicone phantom model mimicking actual brain vascular vessels. Transparent silicone was selected as the vascular material, with an inner diameter of 3.5 mm and a wall thickness ranging from 0.25 to 0.5 mm. The curved segment of the model measured 15.88 mm in length, with a radius (*R* value) of 3.57 mm. Access ports were created in the phantom model at the middle cerebral artery, carotid artery, and vertebral artery to facilitate fluid flow and blood clot loading. The working fluid was composed of a blend of water (60% by weight) and glycerol (40% by weight) to achieve a density and dynamic viscosity that closely approximate those of blood (1.09 ± 0.03 g/ml and 3.98 ± 0.14 cP, respectively) at an operating temperature of 22.2 °C [28]. The experiments were conducted with a steady inlet flow rate, ensuring optimal conditions for simulating physiological conditions. To confirm the integrity of the phantom model, we assessed the device’s wall apposition using commercially available central circulation thrombectomy catheters. The evaluation ensured that the thrombectomy device maintained consistent contact with the vessel wall, effectively adapting to its curvature and demonstrating excellent wall apposition while retaining a cylindrical shape.

### Preparation of thrombus

For the in-vitro evaluation of thrombus removal performance, we synthesized a polymer hydrogel to create an artificial thrombus model. We synthesized thrombi based on polymer hydrogels with two different properties: hard and soft types, following the method applied in previous studies [29, 30]. In brief, Polyacrylamide and alginate (PAAM-Alg) with a crosslinking agent were utilized. The composition for hard thrombus consisted of 88.9% PAAM and 85% solvent (100 wt% DI water), while soft thrombus comprised 88.9% PAAM and 93% solvent (100 wt% DI water). In this study, the hard clot frequently fractured during the testing process, whereas the soft clot exhibited physical properties more similar to actual thrombi, thereby facilitating the evaluation process. For the in vitro clot removal test, the synthesized clot model was cut to a diameter of 3 mm and a length of 20 mm for injection into the vascular model. The clot was inserted into the vascular model through an 8F guide catheter (Terumo, Tokyo, Japan) placed proximally at the target site, which had a diameter of 2–3 mm. The embedding time of the clot was set at 15 min, allowing it to settle in the target site, and the stent retriever was deployed across the clot through a 0.021-in microcatheter (Prowler Select Plus, Johnson & Johnson MedTech, USA). After 5 min of embedding time, the stent retriever and microcatheter were retrieved together into the guide catheter. The thrombus retrieval rate was evaluated as the percentage of pre-intervention to post-intervention clot weight.

## Data Availability

The datasets generated and/or analysed during the current study are not publicly available due [REASON WHY DATA ARE NOT PUBLIC] but are available from the corresponding author on reasonable request.
